# Supplementary material for: Empirical and model-based evidence for a negligible role of cattle in peste des petits ruminants virus transmission and eradication
Source: Commun Biol. 2024 Aug 3;7:937. doi: 10.1038/s42003-024-06619-2 (PMC11297268; doi:10.1038/s42003-024-06619-2)
Supplement: Supplementary file 2 — Supplemental Information [file 42003_2024_6619_MOESM2_ESM.pdf]

Supplementary Information for
Empirical and model-based evidence for a negligible role of cattle in peste des petits ruminants virus transmission and eradication

Catherine M. Herzog[1,#*], Fasil Aklilu[2,#], Demeke Sibhatu[2], Dereje Shegu[2], Redeat Belaineh[2], Abde Aliy Mohammed[2], Menbere Kidane[2], Claudia Schulz[3], Brian J. Willett[4], Sarah Cleaveland[5], Dalan Bailey[6], Andrew R. Peters[8], Isabella M. Cattadori[1], Peter J. Hudson[1], Hagos Asgedom[2], Joram Buza[7], Mesfin Sahle Forza[2,‡], Tesfaye Rufael Chibssa[2], Solomon Gebredufe[2], Nick Juleff[9], Ottar N. Bjørnstad[1], Michael D. Baron[6], Vivek Kapur[1*]

Catherine M. Herzog, Vivek Kapur
Email: cqh5447@psu.edu, vkapur@psu.edu

This PDF file includes:

Supplementary text S1 to S6
Table S1 to S2
Figures S1 to S16

Other supplementary materials for this manuscript include the following:

None

Supplementary Information Text

Text S1: Trial 1 and 2 Detailed Results

Local PPRV isolate produced expected clinical disease in sheep and goats and transmitted from sheep to sheep and goat to goat

Trials 1 and 2 (Figures S4-S5) established that a single 1 mL intranasal dose of the PPRV/Ethiopia/Habru/2014 isolate produced typical clinical signs in local breeds of sheep and goats including fever, conjunctivitis, severe ocular and nasal discharge, diarrhea, altered respiration and coughing, changes in behavior (increasingly inactive, inappetence), and death from infection (trial 2). In both trials, rectal temperature in sheep was higher than that of goats throughout the trial. A modified clinical score (no rectal temperature) removed the difference in the rise and peak of clinical score between sheep and goats (Figure S6), but sheep still had a significantly different and slower return to baseline clinical score than goats (evidenced by non-overlapping confidence intervals), driven by Trial 2 sheep observations. qRT-PCR peak Ct values for swabs generally occurred after peak rectal temperature and before or coinciding with peak clinical score (Figure S4), except for viremia in whole blood which peaked at approximately the same time as peak rectal temperature for inoculated and sentinel sheep but appeared delayed for inoculated goats in both trials (Figures S4-S5). This delay is likely an artifact of the LOESS smooth, and a closer inspection of combined Trial 1 and 2 whole blood data and individual trajectories revealed a biphasic pattern in both sheep and goats (Figure S7). As the pattern of viremia in whole blood was different to that seen in swabs, and as the important measure for this study was the excretion of PPR virus (as shown in swabs), whole blood was not assayed for PPRV RNA after Trial 2. In trial 1, one goat was euthanized due to symptom severity and one control animal died at the start of the trial due to other causes.

The isolate transmitted readily from sheep to sheep and from goat to goat (Trial 2). The co-housed animals all developed pyrexia and clear clinical signs with peak values for rectal temperature and clinical score at 10-12 and 7-11 days, respectively, after the corresponding peak for the inoculated animals. Similarly, seroconversion was detected by 7 dpi for inoculated animals and by 17 dpi for sentinel animals (Figure S5). The lag observed for measures of virus excretion (qRT-PCR) was shorter (5-9 days for sheep, 1-6 days for goats; NB: LOESS smooth did not capture first goat peak in whole blood well so modified range for all goat measures is likely 4-7 days). PPRV was isolated in cell culture from swabs collected on 4, 7, 10 dpi from inoculated animals in trials 1 or 2 (Figure S4 and S5, red crosses) and on 10, 14, and 17 dpi from co-housed animals (trial 2, Figure S5, red circles). All the co-housed animals were infected with PPRV and developed the same level of disease seen in the inoculated animals. When comparing antigen ELISA and qRT-PCR results from nasal swabs taken from both inoculated and co-housed sheep and goats, findings were comparable in trajectory, dpi of peak value, inter-peak interval in days, and uncertainty (Figure S8).

Challenge experiment and controls confirm expected disease progression and facility biosecurity

Seropositive and seronegative goats remaining from trial 3 were challenged with PPRV 13 days after the end of trial 3. The 3 PPRV-seropositive goats were placed in the same barn and were all protected from disease upon challenge. The 7 seronegative goats were not moved and developed clinical signs of PPR and seroconverted upon challenge (Figure S9). No virus isolation data were collected during the challenge experiment and no animals died.

Positive (inoculated) and negative (PPRV seronegative) control animals (see Methods) from each trial were run at the same time in a randomly assigned barn. The results from these control animals in trials 1-2 (Figures S4-S5) and trials 3-5 (Figures S10-12) were as expected with the positive controls developing PPR (clinical signs, seroconversion, RNA shedding) and negative controls showing no detectable clinical signs of PPR or seroconversion.

Text S2: Supplemental Methods

Virus

The PPRV lineage IV (LIV) isolate was stored as a pooled sample of nasal and ocular swabs and gum debris according to Alemu et al.

Animals and Study Design

Markets animals were purchased from were in Western and Northern Showa and included Muger (Inchiny), Ginchi-Dendi, Ejere, and Degem. Sellers reported bringing animals to market from within a 20 km radius Animals were housed on the AHI campus and baseline serological testing for PPRV was conducted to confirm seronegative status. Water and hay were provided ad libitum and refreshed daily. On each day, animals were fed, the barn cleaned of old feed and waste, the rectal temperatures of all animals was determined, and animals were monitored for clinical signs.

All animals were infected intranasally using a 3mL syringe fitted with a nasal atomizer (MAD-300, Dixie EMS Supply). Nasal, ocular, and rectal swabs were collected in duplicate and sent directly to the molecular and cell culture laboratories for processing. Swabs were collected using dry cotton or synthetic swabs and directly soaked in 1 ml of DMEM with 2% fetal bovine serum (FBS), and 1x (ocular and nasal swab) or 5x (rectal swab) antibiotic and antimycotic (Corning: 10,000 units/ml penicillin, 10mg/ml streptomycin sulfate, 25 µg/ml amphotericin B combination, VWR 45000-616).

Negative controls were used in each trial to have comparative rectal temperature, clinical signs, and serology data and assess biosecurity practices to ensure PPRV was not spreading between barns. Positive controls were used to check for expected transmission between small ruminants or expected clinical signs from isolate passage used. To reduce the number of animals used, the number of controls was reduced over the course of the five trials. For Trials 1-2 there were 3 each of sheep and goat negative controls. For Trial 3 there were positive controls (4 inoculated, 4 sentinel goats), and 3 each of cattle and goat negative controls. For Trial 4 there were positive controls (2 inoculated, 2 sentinel goats), and 2 goat negative controls. For Trial 5 there were 2 inoculated goat positive controls and 2 goat negative controls.

Serological and Molecular Analysis

Serological analysis was conducted using the ID Screen PPR Competition kit (IDvet PPRC-4P and PPRC-10P, France).

For molecular analysis, RNA was extracted using the QIAamp Viral RNA Mini kit (Qiagen 52904 and 52906), and real time quantitative RT-PCR conducted with custom Taqman QSY probe (Thermofisher 4482777, FAM-5-CACCGGAYACKGCAGCTGACTCAGAA- 3-QSY) and Express One Step kit (Thermofisher 1178101K) on an Applied Biosystems 7500 FAST instrument. The forward and reverse primers of the PPRV N gene were 5-AGAGTTCAATATGTTRTTAGCCTCCAT-3 and 5-TTCCCCARTCACTCTYCTTTGT-3, respectively. To observe molecular dynamics during the trials, swabs were tested by sandwich ELISA (ID Screen PPR Antigen Capture, IDvet PPRAG-2P, France). Antigen testing with this kit could be run more rapidly after sampling than qRT-PCR testing, which was typically conducted after a trial was complete. Antigen results from Trial 2 (Fig S8), the post Trial 3 challenge (Fig S9), all cattle-to-goat transmission trial barns (Fig S13), and positive control barns in Trials 3-5 (Figs S10-12) were visualized.

Virus Isolation

VDS cells were grown under selection with Zeocin (at 100 µg/ml every third passage; Alfa Aesar Thermofisher Scientific: 100 mg/ml in HEPES, VWR AAJ67140-XF) in DMEM growth media (Corning: VWR 45000-316) containing 2% fetal bovine serum (Avantor Seradigm Select Grade FBS, USDA approved origin, heat-inactivated, VWR 89510-188), supplemented with L-glutamine (Gibco: Glutamax 100x, Thermofisher 35050) and 1:100 antibiotic and antimycotic preparation (Corning: 10,000 units/ml penicillin, 10mg/ml streptomycin sulfate, 25 µg/ml amphotericin B combination, VWR 45000-616).

Text S3: Experimental Settings
Facility
The experimental facility has six experimental barns inside the facility, three on each side of a central hallway (Figure S3). Each barn had an enclosed, small courtyard (approximately 4 meters by 3 meters) with no roof and a closed, roofed barn (approximately 8 meters long, 3 meters wide) with skylight and wire mesh ventilation that allowed air to enter the front or back, but not between barns. During acclimatization the animals were allowed to roam in their assigned barn courtyard (no roof) and inside their assigned roofed barn; however, upon the start of the trial, animals were solely kept inside the roofed barns. The maximum number of animals in a barn was 8 (4 inoculated, 4 co-housed PPRV seronegative animals). Water and feed were provided ad libitum and refreshed daily and the barn was cleaned of debris and fecal material. Specifically, a half bail of hay (6kg) and 2kg concentrate per calf per day and 3kg hay and 1 kg concentrate per sheep/goat per day. From the second trial onward, the barn-length feeding trough had metal bars to discourage sheep and goats from jumping into feeding trough. In cold weather, grass was added for bedding and drafts around the door were blocked with additional boards.

Biosecurity
All personnel entering the facility, including animal attendants, were required to shower to enter and exit the facility (Figure S3A). All personnel wore boots, scrubs, a full body Flexothane suit (Sioen Montreal coverall 4964A2FC1), and N95 masks (masks starting in Trial 2, March 2020) for use solely on this project while in the facility. A footbath was present to be used upon entry and exit just after the showers and at the entrance to the central hallway. For each barn (Figure S3B) there were two footbaths, one just inside the door of the courtyard from the central hallway and one just in front of the door to the roofed barn and all personnel stepped in both baths on the way in or out of the barns. Each animal attendant was assigned to a specific barn and did not enter other barns when a trial was actively running, whereas the sampling team of veterinarians and researchers (typically 3 or less) moved between barns for sampling, which started in the morning and ended by early afternoon. In total, to enter or exit a particular barn, personnel stepped in 4 footbaths filled with a dilution of FAM-30 disinfectant (Evans Vanodine, R067 KEV). When exiting any barn, while stepping in the first footbath, the sampling team and barn-specific attendants assisted each other to spray and sponge down their Flexothane suits with FAM-30 dilutions available in spray bottles. They waited for the suits to dry before entering the courtyard of the next barn. Clinical signs data was recorded by photo of a whiteboard in each barn and of each animal (photos included: eartag, head profile, frontal profile, conjunctival tissue, oral view, rectal view). A Go-Pro Hero 7 camera in waterproof housing case, used to record photos and video of clinical data and signs in the barn, was also sprayed with FAM-30 disinfectant on the way out of each barn, as were any items leaving the barn with the sampling team. Each barn had its own supply of latex gloves, biohazard trash bags, trash container, whiteboard makers and erasers, sample labelling markers, and broom that stayed within the barns. New gloves were put on by the personnel once they arrived in each barn and these gloves were discarded before leaving a barn. Prepared racks with labeled blood and serum vials and cryovials for collecting molecular swabs where only brought into the assigned barns, sprayed with FAM-30 upon exit, and stored in a cooler with icepacks while sampling was completed. They were then transferred immediately to AHI sample reception and appropriate laboratories. At the end of a trial, barns were cleaned of feed and waste and were sprayed down with FAM-30 disinfectant and left to dry before moving on to the next trial. Each barn had its own drain in the floor to take liquid waste to a tank on the side.

Text S4. Power analysis details to determine detectable probability of cattle-to-goat transmission

Transmission is a binary event that can be modeled by binomial distribution Bin(n, p) where the distribution is the number of successes (transmissions) in n number of independent trials with a probability p of success (transmission) for each trial. In this trial there were 32 cattle goat pairs (trials). We simulated 1000 trial outcome observations, summing the outcomes and divided by 1000 to obtain an average, simulated a range of probabilities of cattle-to-goat transmission (we used 0.0001 to 0.1 or 0.01-10%), and plotted these values. We drew a horizontal line on the y axis at 0.8 – indicating the cutoff where there was an 80% chance of detecting cattle-to-goat transmission, if it occurred. The vertical line intersects the simulated data at a probably of cattle-to-goat transmission of 5%, which is the lowest probability of transmission this study is powered to detect.

Figure S1 can be recreated by running the following R code. Comments provided for clarity:

```
# creates an empty vector of length 100 filled with NA. this vector will hold the y axis values, the probability of detecting transmission.
q <- rep(NA,100);

# creates a vector of length 100 filled with a sequence of numbers to represent low probability values which ranged between 0.0001 and 0.1. this vector will hold the x-axis values, the probability of C->G transmission.
pp <-seq(0.0001,0.1,length = 100);

# loops over the 100 indexed spots in the q vector and generates random draws from a binomial distribution (1000 observations of 32 trials with probabilities greater than 0 given in the pp vector), which are summed and divided by 1000 to generate an average value, and the average value is placed into the respective spot in the empty q vector)
for(i in 1:100){q[i] <- sum(rbinom(1000,32,pp[i])>0)/1000}

# plots pp and q vector and adds text for x and y axes
plot(pp,q, xlab = "Probability of C -> G Transmission", ylab= "Probability of Detecting")

# adds vertical and horizontal lines to the plot
abline(v=0.05)
abline(h=0.8)
```

Text S5. R code for estimation of the posterior distributions for cattle-to-goat, goat-to-cattle, and goat-to-goat transmission probabilities and their corresponding ratesconfidence distribution based on experimental results

```
library(bayesrules)
library(dplyr)
library(ggplot2)

set.seed(84735)

# transmission probability C->G
# the prob that an exponentially distributed waiting time <  trial length (35 days)
# convert posterior mean from probability to rate using p(t) = 1-e^-rt or -ln(1-p(t))/t = r
plot_beta(1, 1) # visualize uninformative prior (uniform - special case of beta)
plot_beta_binomial(alpha = 1, beta = 1, y = 0, n = 32) # look at prior and likelihood based on experimental results
summarize_beta_binomial(alpha = 1, beta = 1, y = 0, n = 32)
post_mean_ctg <- summarize_beta_binomial(alpha = 1, beta = 1, y = 0, n = 32)[2,4]  # pull posterior mean from summaries of prior and posterior distribution
# rbeta(alpha + y, beta + n - y), here rbeta(1 + 0, 1 + 32 - 0) or rbeta(1,33) - the distribution the beta binomial is summarizing
qbeta(c(.025, 0.975), 1, 33) # 95% credible interval for C->G transmission probability
# 0.0007669121 0.1057628101

# Calculating rates
# 14d
# beta_rate_ctg = -log(1-post_mean_ctg)/14 # convert mean probability to a rate during the 35 day period of the C->G trials
# beta_rate_ctg # 0.002132355
# sample_ctg <- rbeta(10000, 1, 33)
# rates_ctg <- -log(1-sample_ctg)/14
# quantile(rates_ctg, probs = c(.025, 0.975))
# # 2.5%         97.5%
# # 0.0000491082 0.0081290883

#10d
beta_rate_ctg = -log(1-post_mean_ctg)/10 # convert mean probability to a rate during the 35 day period of the C->G trials
beta_rate_ctg # 0.002985296
sample_ctg <- rbeta(10000, 1, 33)
rates_ctg <- -log(1-sample_ctg)/10
quantile(rates_ctg, probs = c(.025, 0.975))
# 2.5%       97.5%
# 8.59845e-05 1.12721e-02

#8d
# beta_rate_ctg = -log(1-post_mean_ctg)/8 # convert mean probability to a rate during the 35 day period of the C->G trials
# beta_rate_ctg # 0.00373162
# sample_ctg <- rbeta(10000, 1, 33)
# rates_ctg <- -log(1-sample_ctg)/8
# quantile(rates_ctg, probs = c(.025, 0.975))
# # 2.5%          97.5%
# # 9.777366e-05  1.461837e-02
```

```
# transmission probability G->G
plot_beta(1, 1)
plot_beta_binomial(alpha = 1, beta = 1, y = 12, n = 12)
summarize_beta_binomial(alpha = 1, beta = 1, y = 12, n = 12)
post_mean_gtg <- summarize_beta_binomial(alpha = 1, beta = 1, y = 12, n = 12)[2,4]
# rbeta(alpha + y, beta + n - y), here rbeta(1 + 12, 1 + 12 - 12) or rbeta(13,1) - the
distribution the beta binomial is summarizing
qbeta(c(.025, 0.975), 13, 1) # 95% credible interval for C->G transmission probability
# 0.7529474 0.9980544

# Calculating rates
# # 14d
# beta_rate_gtg = -log(1-post_mean_gtg)/14
# beta_rate_gtg #0.1885041
# sample_gtg <- rbeta(10000, 13, 1)
# rates_gtg <- -log(1-sample_gtg)/14
# quantile(rates_gtg, probs = c(.025, 0.975))
# # 2.5%      97.5%
# # 0.1005720 0.4435633

# 10d
beta_rate_gtg = -log(1-post_mean_gtg)/10
beta_rate_gtg #0.2639057
sample_gtg <- rbeta(10000, 13, 1)
rates_gtg <- -log(1-sample_gtg)/10
quantile(rates_gtg, probs = c(.025, 0.975))
# 2.5%      97.5%
# 0.1437850 0.6335271

# # 8d
# beta_rate_gtg = -log(1-post_mean_gtg)/8
# beta_rate_gtg #0.3298822
# sample_gtg <- rbeta(10000, 13, 1)
# rates_gtg <- -log(1-sample_gtg)/8
# quantile(rates_gtg, probs = c(.025, 0.975))
# # 2.5%      97.5%
# # 0.1771663 0.7814859



# transmission probability G->C
plot_beta(1, 1)
plot_beta_binomial(alpha = 1, beta = 1, y = 2, n = 2)
summarize_beta_binomial(alpha = 1, beta = 1, y = 2, n = 2)
post_mean_gtc <- summarize_beta_binomial(alpha = 1, beta = 1, y = 2, n = 2)[2,4]
# rbeta(alpha + y, beta + n - y), here rbeta(1 + 2, 1 + 2 - 2) or rbeta(3,1) - the distribution the
beta binomial is summarizing
qbeta(c(.025, 0.975), 3, 1) # 95% credible interval for C->G transmission probability
# 0.2924018 0.9915962

# Calculating rates
# # 14d
# beta_rate_gtc = -log(1-post_mean_gtc)/14
# beta_rate_gtc # 0.09902103
# sample_gtc <- rbeta(10000, 3, 1)
```

```
# rates_gtc <- -log(1-sample_gtc)/14
# quantile(rates_gtc, probs = c(.025, 0.975))
# # 2.5%       97.5%
# # 0.02467719 0.33366332

# 10d
beta_rate_gtc = -log(1-post_mean_gtc)/10
beta_rate_gtc # 0.1386294
sample_gtc <- rbeta(10000, 3, 1)
rates_gtc <- -log(1-sample_gtc)/10
quantile(rates_gtc, probs = c(.025, 0.975))
# 2.5%     97.5%
# 0.034547 0.487640

# # 8d
# beta_rate_gtc = -log(1-post_mean_gtc)/8
# beta_rate_gtc # 0.1732868
# sample_gtc <- rbeta(10000, 3, 1)
# rates_gtc <- -log(1-sample_gtc)/8
# quantile(rates_gtc, probs = c(.025, 0.975))
# # 2.5%       97.5%
# # 0.04376008 0.58085127


# Comparison of G->G vs C->G transmission rate
beta_rate_gtg/beta_rate_ctg
# 88.40186  Goats have a ~88x greater transmission rate to other goats than cattle to goats

# Comparison of G->G vs G->C
beta_rate_gtg/beta_rate_gtc
# 1.903677
```

Text S6. R code for modeling framework

```
# Empirical and model-based evidence for a negligible role of cattle in
# peste des petits ruminants transmission and eradication

# Catherine M. Herzog#*, Fasil Aklilu#, Demeke Sibhatu, Dereje Shegu,
# Redeat Belaineh, Abde Aliy Mohammed, Menbere Kidane,  Claudia Schulz,
# Brian J. Willett, Sarah Cleaveland, Dalan Bailey, Andrew R. Peters,
# Isabella M. Cattadori, Peter J. Hudson, Hagos Asgedom, Joram Buza,
# Mesfin Sahle Forza, Tesfaye Rufael Chibssa, Solomon Gebredufe, Nick Juleff,
# Ottar N. Bjørnstad, Michael D. Baron, Vivek Kapur*

# Code for: 2 species SIR model with vaccination + transmission asymmetries
# Code authors: Ottar N. Bjørnstad + Catherine Herzog

######################################################
# Next Generation Matrix function & example usage
######################################################
require(deSolve)

nextgenR0=function(Istates, Flist, Vlist, params, dfe){
  paras = as.list(c(dfe, params))

  k=0
  vl=fl=list(NULL)
  for(i in 1:length(Istates)){
    assign(paste("f", i, sep = "."), lapply(lapply(Flist,deriv, Istates[i]), eval, paras))
    assign(paste("v", i, sep = "."), lapply(lapply(Vlist,deriv, Istates[i]), eval, paras))
    for(j in 1:length(Istates)){
      k=k+1
      fl[[k]]=attr(eval(as.name(paste("f", i, sep=".")))[[j]], "gradient")[1,]
      vl[[k]]=attr(eval(as.name(paste("v", i, sep=".")))[[j]], "gradient")[1,]
    }
  }

  f=matrix(as.numeric(as.matrix(fl)[,1]), ncol=length(Istates))
  v=matrix(as.numeric(as.matrix(vl)[,1]), ncol=length(Istates))
  R0=max(eigen(f%*%solve(v))$values)
  return(R0)
}


# Recipe for use
# Step 1: All states
istates=c("I1", "I2")

# Step 2: All new infections:
flist=c(dI1dt=quote(beta11 * S1 *(1-p1) * I1 / N1 + beta21 * S1 *(1-p1)* I2 / N1),
dI2dt=quote(beta12 * S2*(1-p2) * I1 / N2 + beta22 * S2*(1-p2)* I2 / N2))

#Step 3-5
#All losses
Vm1=quote(mu1 * I1 + gamma1 * I1)
Vm2=quote(mu2 * I2 + gamma2 * I2)
#All gained transfers$
Vp1=0
```

```
Vp2=0
#Subtract Vp from Vm
V1=substitute(a-b, list(a=Vm1, b=Vp1))
V2=substitute(a-b, list(a=Vm2, b=Vp2))
#Make Vlist
vlist = c(V1,V2)

#Define list of parameter vectors:
# This is done for each figure below

#Specify disease-free equilibrium
df = list(S1 = 1, S2 = 1, I1 = 0, I2 = 0)

#Call nextgen function


##########################################
# Figure 3 usage
##########################################

# Figure Margin Setup
# set working directory
par(mfrow = c(2,2),
    oma = c(4,6.5,0,1) + 0.1,
    mar = c(1,2,1,1) + 0.1)
#jpeg("Figure3.jpeg", width = 12, height = 10, units = "in", quality = 100, res = 600)

# # Transmission rates - include rates from only 1 infectious period per run of this script
# # 14 day infectious period
# beta_rate_ctg = 0.002132355  # (n=32 goats)
# beta_rate_ctc = 0.002132355  # no empirical data, have assumed same as C->G
# beta_rate_gtg = 0.1885041    # (n=12 goats)
# beta_rate_gtc = 0.09902103    # (n=2 cattle)

# 10 day infectious period
beta_rate_ctg = 0.002985296  # (n=32 goats)
beta_rate_ctc = 0.002985296  # no empirical data, have assumed same as C->G
beta_rate_gtg = 0.2639057    # (n=12 goats)
beta_rate_gtc = 0.1386294    # (n=2 cattle)

# # 8 day infectious period
# beta_rate_ctg = 0.00373162  # (n=32 goats)
# beta_rate_ctc = 0.00373162  # no empirical data, have assumed same as C->G
# beta_rate_gtg = 0.3298822    # (n=12 goats)
# beta_rate_gtc = 0.1732868    # (n=2 cattle)

#Mortality rates (converted to daily rates)
# Small ruminant rate from literature. Inverse of rate gives life span ~ 2.15 years.
# Yitagesu et al 2022 # https://www.ncbi.nlm.nih.gov/pmc/articles/PMC9514490
# Cattle mortality rate order of magnitude smaller than small ruminants; inverse gives 10
year life span
mu_s = ((0.629+.302)/2)/365 # mortality rate (mean of kids and adults) per animal year
mu_c = .10/365

# Recovery rate
# Recover usually occurs by ~ 14 days
```

```
gamma_s = 1/14
gamma_c = 1/14

########################################
# Symmetric spill forward and spillback
########################################

parms  = list(mu = c(mu_s, mu_c),
              N = c(1,1),
             beta =  matrix(c(beta_rate_gtg, beta_rate_gtg, beta_rate_gtg, beta_rate_gtg),
              ncol=2, byrow=TRUE),
              gamma = c(gamma_s, gamma_c),
              p=c(0, 0))
#Extract to work with nextgenR0:
para = list(mu1 = parms$mu[1],
            mu2 = parms$mu[2],
            beta11 = parms$beta[1,1],
            beta12 = parms$beta[1,2],
            beta21 = parms$beta[2,1],
            beta22 = parms$beta[2,2],
            gamma1 = parms$gamma[1],
            gamma2 = parms$gamma[2],
            p1 = parms$p[1],
            p2 = parms$p[2],
            N1 = parms$N[1],
            N2 = parms$N[2])

beta2=seq(0, round(beta_rate_gtg, 2), length.out = 101)
p=seq(0,1, by=0.01)
RE=matrix(NA, ncol=length(beta2), nrow=length(p))
for(i in 1:length(beta2)){
  for(j in 1:length(p)){
    para$p1=p[j]
    para$beta21=beta2[i]
    RE[i,j]=nextgenR0(Istates=istates, Flist=flist, Vlist=vlist, params=para, dfe=df)
  }
}

# plot
contour(beta2, p, RE, levels = seq(0,9,1), lwd = 2, vfont = c("sans serif", "bold"), labcex =
1.25,
        #main = "Symmetric",
        xaxt = "n", yaxt = "n", cex.axis = 1.5)
axis(side = 1, at = seq(0,max(beta2), by =0.2), labels = FALSE)
axis(side = 2, at = seq(0,1,by =0.2), labels = c(0, 20, 40, 60, 80, 100), cex.axis = 1.5)

########################################################
# Species Specific Symmetric spill forward and spillback
########################################################

# with 3x more from shoats to cattle than back
parms  = list(mu = c(mu_s, mu_c),
              N = c(1,1),
             beta =  matrix(c(beta_rate_gtg, beta_rate_gtg, beta_rate_ctg, beta_rate_ctg),
              ncol=2, byrow=TRUE),
              gamma = c(gamma_s, gamma_c),
```

```
            p=c(0, 0))
#Extract to work with nextgenR0:
para = list(mu1 = parms$mu[1],
         mu2 = parms$mu[2],
         beta11 = parms$beta[1,1],
         beta12 = parms$beta[1,2],
         beta21 = parms$beta[2,1],
         beta22 = parms$beta[2,2],
         gamma1 = parms$gamma[1],
         gamma2 = parms$gamma[2],
         p1 = parms$p[1],
         p2 = parms$p[2],
         N1 = parms$N[1],
         N2 = parms$N[2])

beta2=seq(0, round(beta_rate_gtg, 2), length.out = 101)
p=seq(0,1, by=0.01)
RE=matrix(NA, ncol=length(beta2), nrow=length(p))
for(i in 1:length(beta2)){
  for(j in 1:length(p)){
    para$p1=p[j]
    para$beta21=beta2[i]
    RE[i,j]=nextgenR0(Istates=istates, Flist=flist, Vlist=vlist, params=para, dfe=df)
  }
}

# plot
contour(beta2, p, RE, levels = seq(0,9,1), lwd = 2, vfont = c("sans serif", "bold"), labcex =
1.25,
      #main = "Species-Specific Symmetric",
      xaxt = "n", yaxt = "n", cex.axis = 1.5)
axis(side = 1, at = seq(0, max(beta2),,by =0.2), labels = FALSE)
axis(side = 2, at = seq(0,1,by =0.2), labels = FALSE, cex.axis = 1.5)




#######################################
#Asymmetric spill forward
#######################################
parms  = list(mu = c(mu_s, mu_c),
            N = c(1,1),
            beta =  matrix(c(beta_rate_gtg, beta_rate_ctg, beta_rate_ctg, beta_rate_ctg),
            ncol=2, byrow=TRUE),
            gamma = c(gamma_s, gamma_c),
            p=c(0, 0))
#Extract to work with nextgenR0:
para = list(mu1 = parms$mu[1],
         mu2 = parms$mu[2],
         beta11 = parms$beta[1,1],
         beta12 = parms$beta[1,2],
         beta21 = parms$beta[2,1],
         beta22 = parms$beta[2,2],
         gamma1 = parms$gamma[1],
         gamma2 = parms$gamma[2],
```

```
            p1 = parms$p[1],
            p2 = parms$p[2],
            N1 = parms$N[1],
            N2 = parms$N[2])

beta2=seq(0, round(beta_rate_gtg, 2), length.out = 101)
p=seq(0,1, by=0.01)
RE=matrix(NA, ncol=length(beta2), nrow=length(p))
for(i in 1:length(beta2)){
  for(j in 1:length(p)){
    para$p1=p[j]
    para$beta21=beta2[i]
    RE[i,j]=nextgenR0(Istates=istates, Flist=flist, Vlist=vlist, params=para, dfe=df)
  }
}

#plot
contour(beta2, p, RE, levels = seq(0,9,1), lwd = 2, vfont = c("sans serif", "bold"), labcex =
1.25,
        #main = "Asymmetric Spill Forward",
        yaxt = "n", cex.axis = 1.5)
axis(side = 2, at = seq(0,1,by =0.2), labels = c(0, 20, 40, 60, 80, 100), cex.axis = 1.5)



########################################
#Asymmetric spill forward and spillback
########################################
parms  = list(mu = c(mu_s, mu_c),
              N = c(1,1),
               beta =  matrix(c(beta_rate_gtg, beta_rate_gtg*(2/3), beta_rate_ctg, 0), ncol=2,
              byrow=TRUE),
              gamma = c(gamma_s, gamma_c),
              p=c(0, 0))
#Extract to work with nextgenR0:
para = list(mu1 = parms$mu[1],
            mu2 = parms$mu[2],
            beta11 = parms$beta[1,1],
            beta12 = parms$beta[1,2],
            beta21 = parms$beta[2,1],
            beta22 = parms$beta[2,2],
            gamma1 = parms$gamma[1],
            gamma2 = parms$gamma[2],
            p1 = parms$p[1],
            p2 = parms$p[2],
            N1 = parms$N[1],
            N2 = parms$N[2])

beta2=seq(0, round(beta_rate_gtg, 2), length.out = 101)
p=seq(0,1, by=0.01)
RE=matrix(NA, ncol=length(beta2), nrow=length(p))
for(i in 1:length(beta2)){
  for(j in 1:length(p)){
    para$p1=p[j]
    para$beta21=beta2[i]
```

```
    RE[i,j]=nextgenR0(Istates=istates, Flist=flist, Vlist=vlist, params=para, dfe=df)
  }
}


#plot
contour(beta2, p, RE, levels = seq(0,9,1), lwd = 2, vfont = c("sans serif", "bold"), labcex =
1.25,
     #main = "Asymmetric Spill Forward & Spillback",
     yaxt = "n", cex.axis = 1.5)
axis(side = 2, at = seq(0,1,by =0.2), labels = FALSE, cex.axis = 1.5)


# Overall plot title and labels
title(xlab = expression(paste("Cattle-to-Goat Transmission Rate (", beta[CS], ")")),
    ylab = "Small Ruminant Population Immunity (%)",
    outer = TRUE,
    line = 3,
    cex.lab = 2)

#invisible(dev.off())
```

Table S1. Concentrations and passage information for isolations used in all experiments.

Trial	Concentration ($TCID_{50}$/ml)	Passage
1	$10^{5.3}$	3rd
2	$10^{5.3}$	3rd
3	$10^{5.6}$	4th
Challenge	$10^{5.6}$	4th
4	$10^{5.5}$	4th
5	$10^{5.5}$	4th

Table S2. Parameter table

Parameter	Description	Scenario 1	Scenario 2	Scenario 3	Scenario 4
β matrix $\beta_{SS}\ \beta_{SC}$ $\beta_{CS}\ \beta_{CC}$	Transmission rate matrix	β_{SS} β_{SC} 0-.26* β_{CC}	β_{SS} β_{SC} 0-.26* β_{CC}	β_{SS} β_{SC} 0-.26* β_{CC}	β_{SS} β_{SC} 0-.26* β_{CC}
β_{SS}	Transmission rate	0.2639057	0.2639057	0.2639057	0.2639057
β_{SC}	Transmission rate	0.2639057	0.2639057	0.002985296	0.1386294
β_{CS}	Transmission rate	0.2639057	0.002985296	0.002985296	0.002985296
β_{CC}	Transmission rate	0.2639057	0.002985296 (assumed)	0.002985296 (assumed)	0
μ_1	Small ruminant demographic rate (births and deaths)**	.4655/365	.4655/365	.4655/365	.4655/365
μ_2	Cattle demographic rate (births & deaths)***	0.1/365	0.1/365	0.1/365	0.1/365
γ_1	Small ruminant recovery rate	1/14	1/14	1/14	1/14
γ_2	Cattle recovery rate	1/14	1/14	1/14	1/14
p_1	Small ruminant vaccination proportion	Varies 0-100%	Varies 0-100%	Varies 0-100%	Varies 0-100%
p_2	Cattle vaccination proportion	0	0	0	0

N_1	Small ruminant population (as density)	1	1	1	1
N_2	Cattle population (as density)	1	1	1	1

(C) = Cattle; (S) Small ruminants
* β_{CS} varies between 0 and 0.26 (from 0 to rate of small ruminant-to-small ruminant transmission for an infectious period of 10 days) as seen in Figure 3.
** Yitagesu et al 2022 reports mortality rate per animal year for kids and adults. We took the mean of these rates and converted to a daily rate. The inverse of this rate yields a lifespan of 2.14 years for small ruminants.
*** The inverse of this rate yields a lifespan of 10 years for cattle.

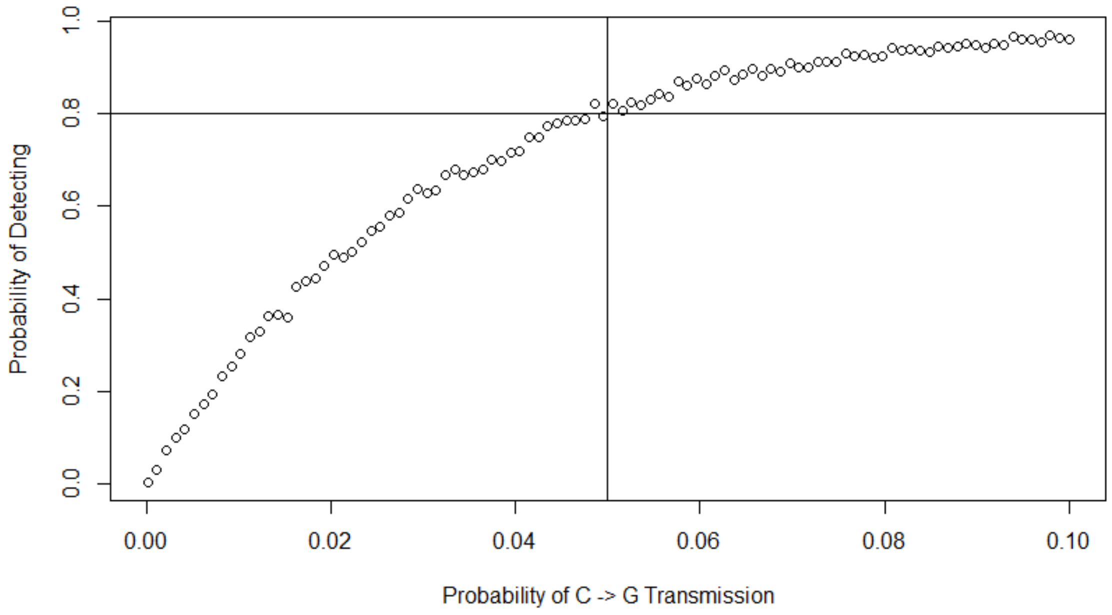

Fig. S1. Power analysis to determine detectable probability of cattle-to-goat transmission. One thousand simulated draws for a range of cattle (C) to goat (G) transmission probabilities (x-axis) were simulated from a binomial distribution with trial size 32. The horizontal line on the y-axis represents a 80% chance of detecting transmission, if it exists. The vertical line intersects the simulated data and horizontal line at a probably of cattle-to-goat transmission of 5%, which is the lowest probability of cattle-to-goat transmission this study was powered to detect.

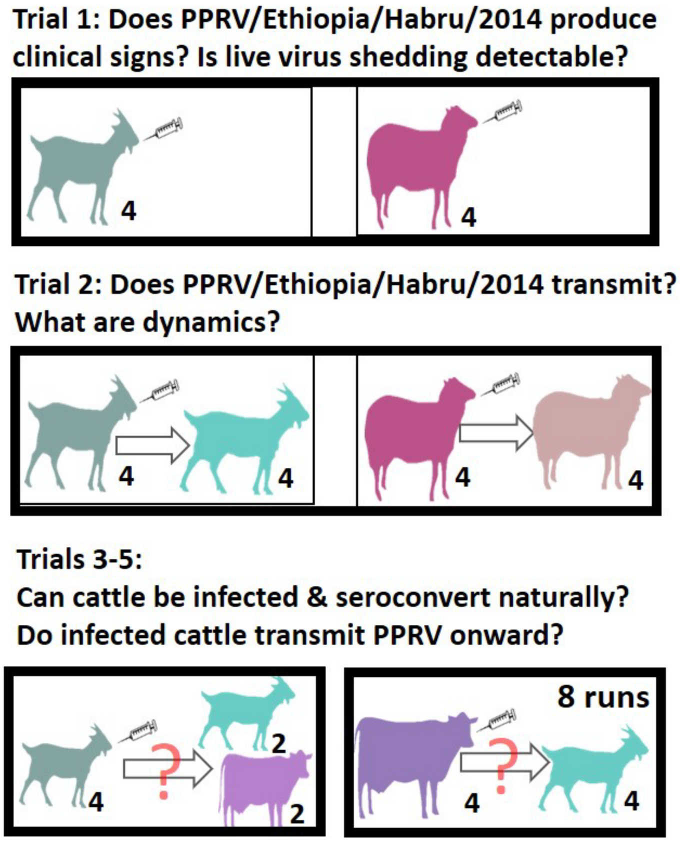

Fig. S2. Study design of Trials 1-5. The series of trials tested that PPRV/Ethiopia/Habru/2014 could infect, cause clinical signs and mortality, and transmit among small ruminants before addressing the open question of cattle spillback transmission to goats directly in Trials 3-5. Controls are not shown but were randomly assigned to one of the six barns in the facility and monitored concurrently with experimental barns during each trial. Darker colored animals with syringes are inoculated, lighter colored animals without syringes are co-housed, PPRV-seronegative animals.

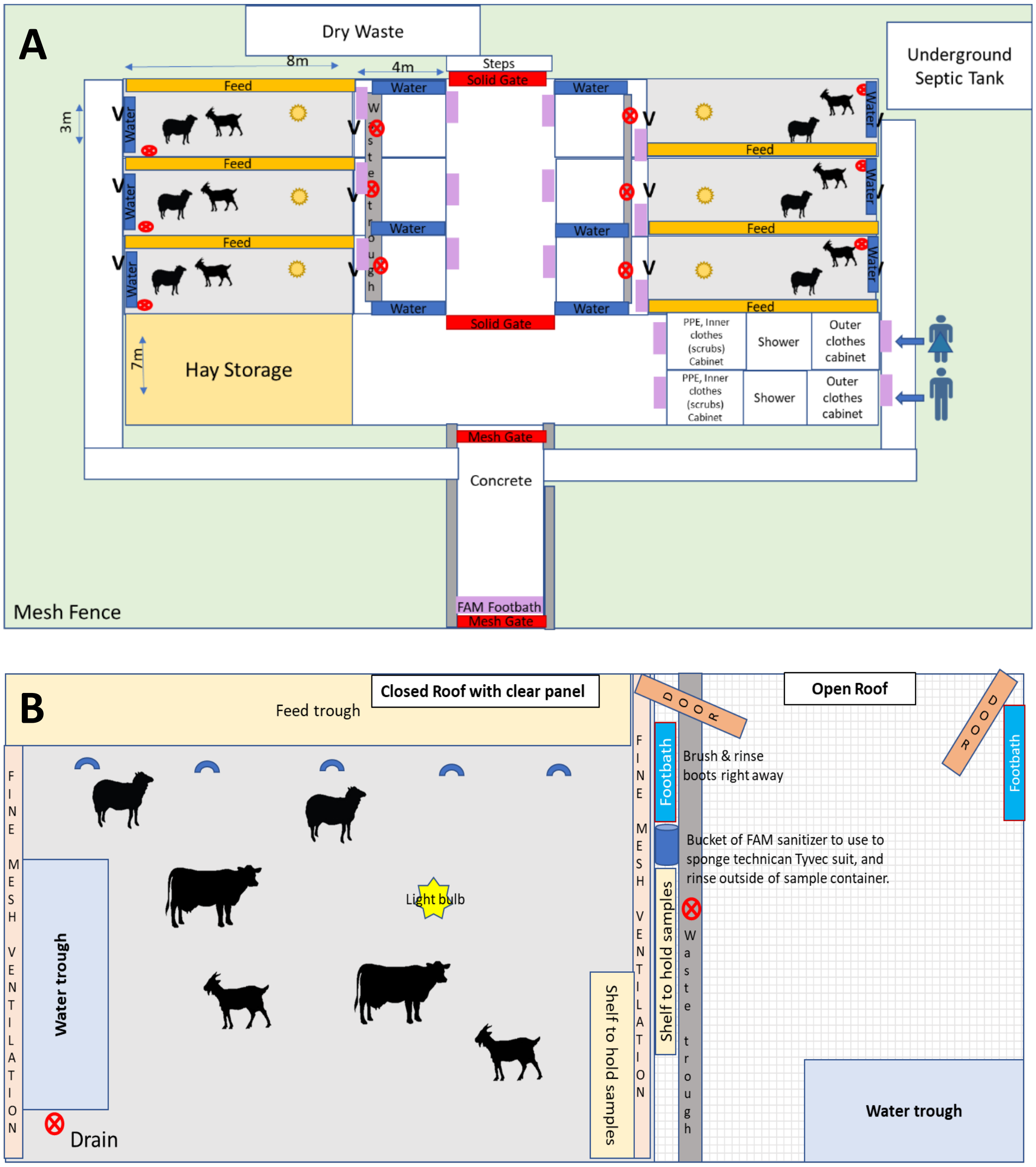

Fig. S3. Animal facility (A) and within barn (B) biosafety and biosecurity workflow.

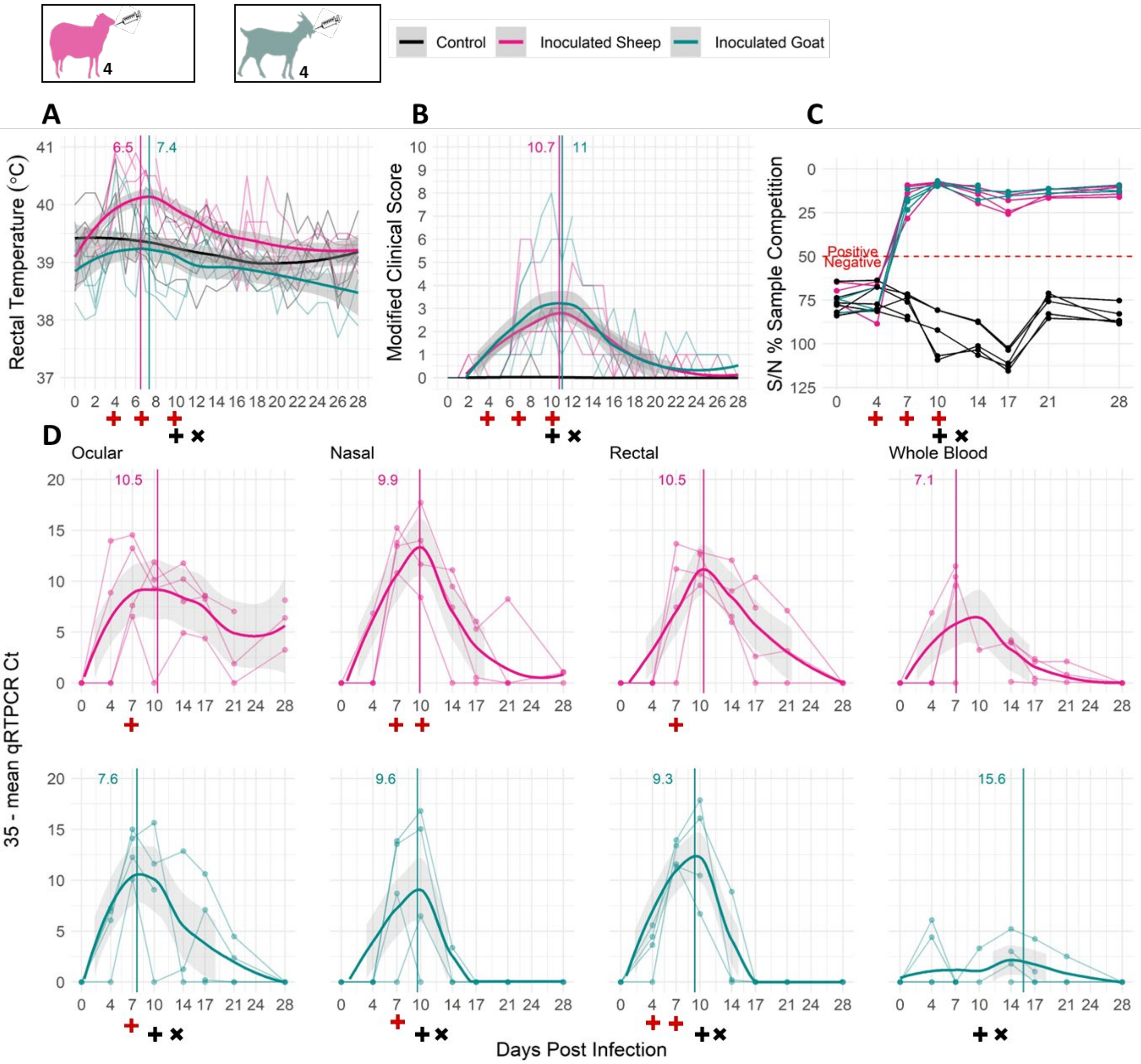

Fig. S4. Infection dynamics of PPRV among local breeds of Ethiopian sheep and goat breeds. Trial 1 **A**. daily rectal temperature, **B**. daily clinical score, **C**. serology (competitive ELISA), and **D**. viral RNA from ocular, nasal, and rectal swabs and whole blood (RT-PCR). Thin lines represent individual animals and bold lines represent smooth local regression (LOESS) curves of all animals in the category (control, inoculated, sentinel). Gray shading indicates 95% confidence bands (t-based approximation). Vertical lines indicate day post infection (dpi) of peak. Sampling days on which PPRV could be isolated (cross if isolated from inoculated animal, circle for sentinel animal) are indicated with red and dpi with deaths are indicated with black (cross for inoculated animal deaths, circle for sentinel deaths, x for euthanized) along the x-axis. Isolation was not conducted on whole blood samples.One inoculated goat was euthanized.

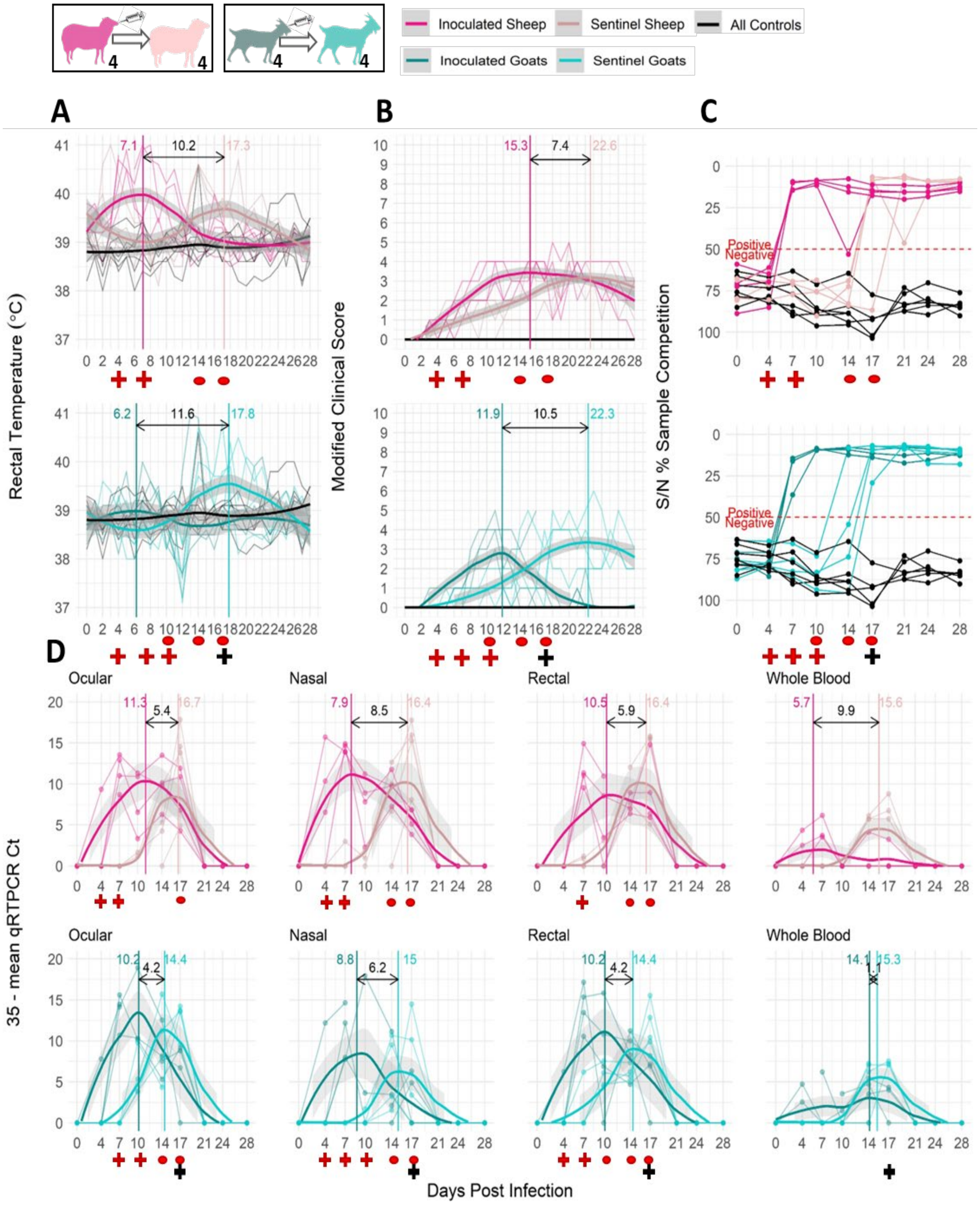
Inoculated Sheep
Sentinel Sheep
All Controls
Inoculated Goats
Sentinel Goats
A
B
C
D
Rectal Temperature (°C)
Modified Clinical Score
S/N % Sample Competition
Positive
Negative
Ocular
Nasal
Rectal
Whole Blood
35 - mean qRTPCR Ct
Days Post Infection

Fig. S5. Within host and transmission dynamics of PPRV among co-housed local breeds of Ethiopian sheep and goats. Trial 2 **A**. daily rectal temperature, **B**. daily clinical score, **C**. serology (competitive ELISA), and **D**. viral RNA from ocular, nasal, and rectal swabs and whole blood (RT-PCR). Thin lines represent individual animals and bold lines represent smooth local regression (LOESS) curves of all animals in the category (control, inoculated, sentinel). Gray shading indicates 95% confidence bands (t-based approximation). Vertical lines indicate day post infection (dpi) of peak value and inter-peak interval (difference in dpi of peak value for each animal group) is indicated in black. Sampling days on which PPRV could be isolated (cross if isolated from inoculated animal, circle for sentinel animal) are indicated with red and dpi with deaths are indicated with black (cross for inoculated animal deaths, circle for sentinel deaths, x for euthanized) along the x-axis. Isolation was not conducted on whole blood samples. One death occurred in an inoculated goat.

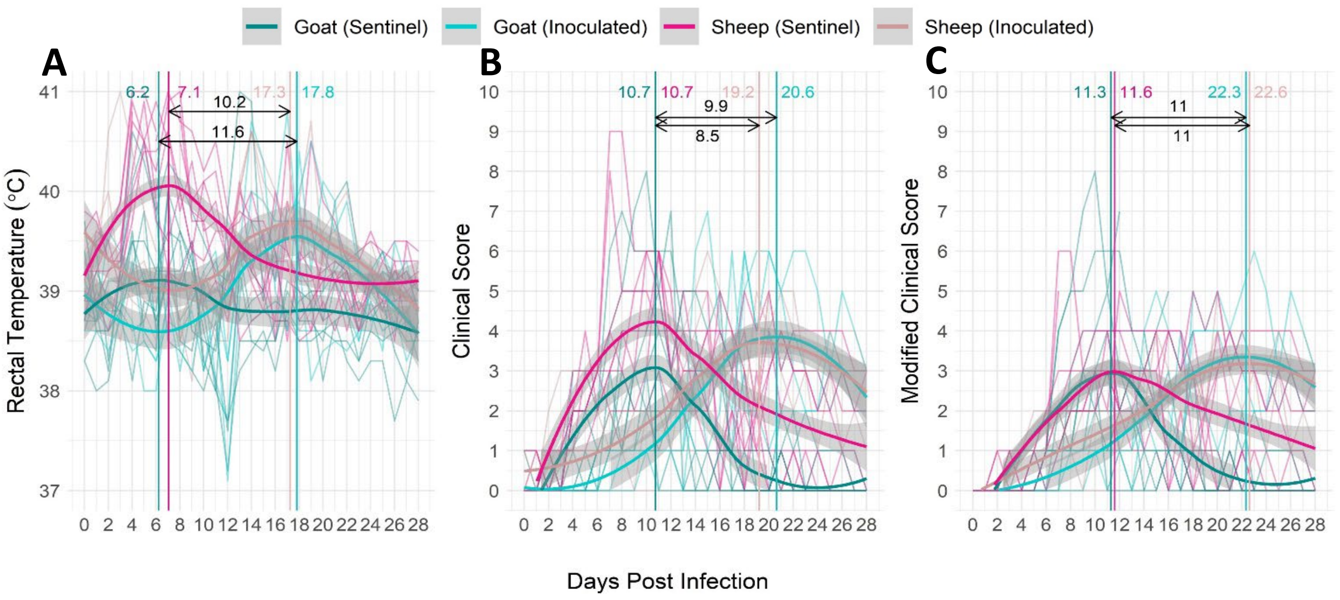

Fig. S6. Impact of rectal temperature (A) on clinical score calculated with (B) and without (C) rectal temperature shows that the rise and peak of clinical score does not differ for sheep and goats, though the return to baseline clinical score may take longer for sheep (C). Combined sheep and goat data from Trials 1 and 2. Thin lines represent individual animals and bold lines represent smooth local regression (LOESS) curves of all animals in the category (inoculated, sentinel). Gray shading indicates 95% confidence bands (t-based approximation). Vertical lines indicate day post infection (dpi) of peak value and inter-peak interval (difference in dpi of peak value for each animal group) is indicated in black.

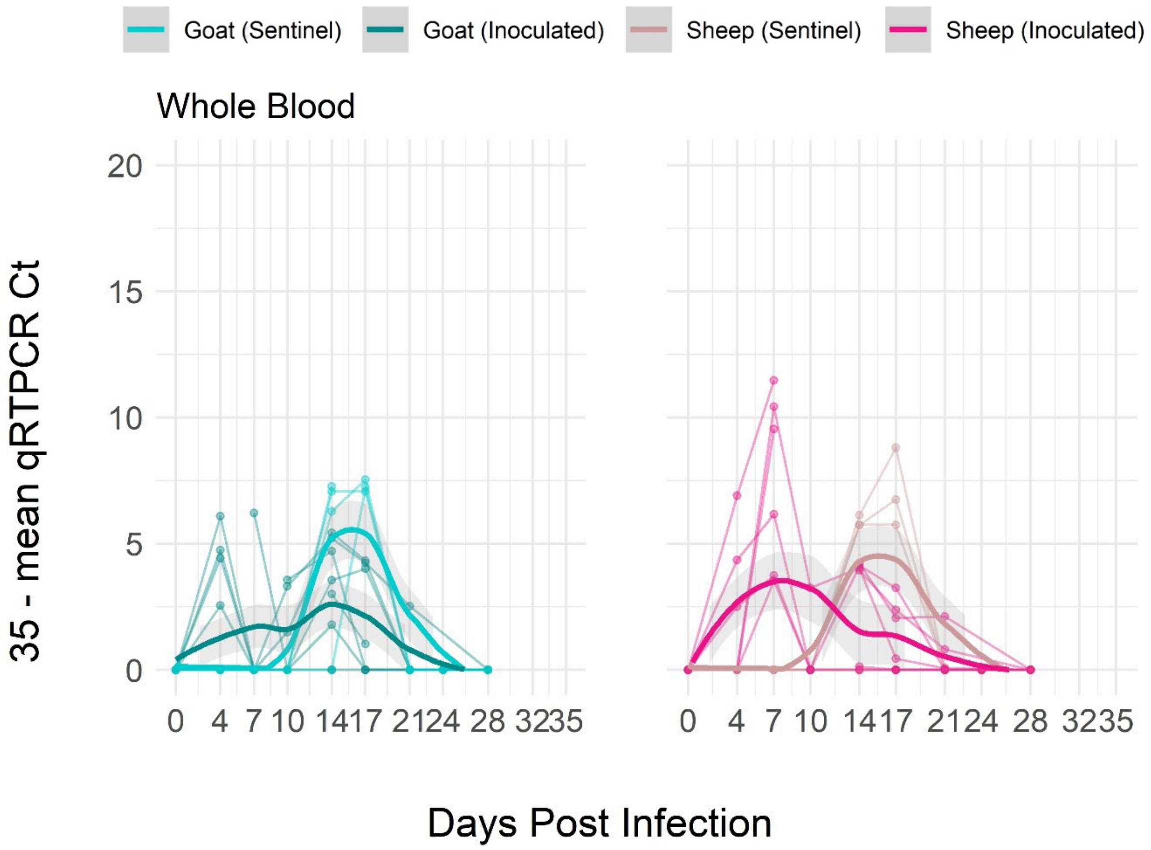

Figure S7. Biphasic pattern in PPRV RNA detected in whole blood samples of sheep and goats in Trials 1 and 2. Thin lines represent individual animals and bold lines represent smooth local regression (LOESS) curves of all animals in the category (inoculated, sentinel). Gray shading indicates 95% confidence bands (t-based approximation).

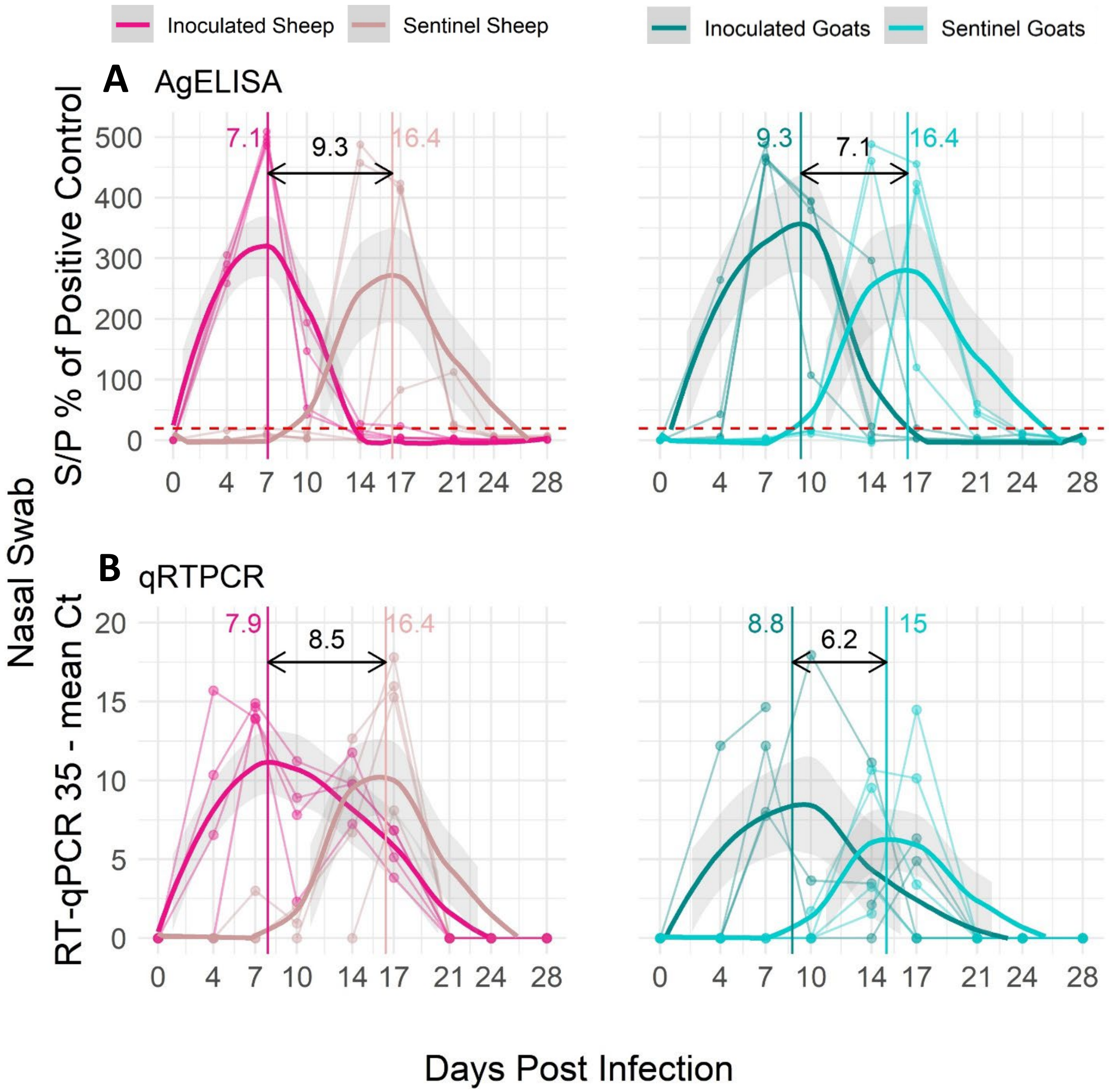

Fig. S8. Comparison of RNA detection between antigen ELISA (A) and RT-PCR (B) among nasal swabs from inoculated and sentinel sheep and goats from Trial 2. Thin lines represent individual animals and bold lines represent smooth local regression (LOESS) curves of all animals in the category (inoculated, sentinel). Gray shading indicates 95% confidence bands (t-based approximation). Vertical lines indicate day post infection (dpi) of peak value and inter-peak interval (difference in dpi of peak value for each animal group) is indicated in black.

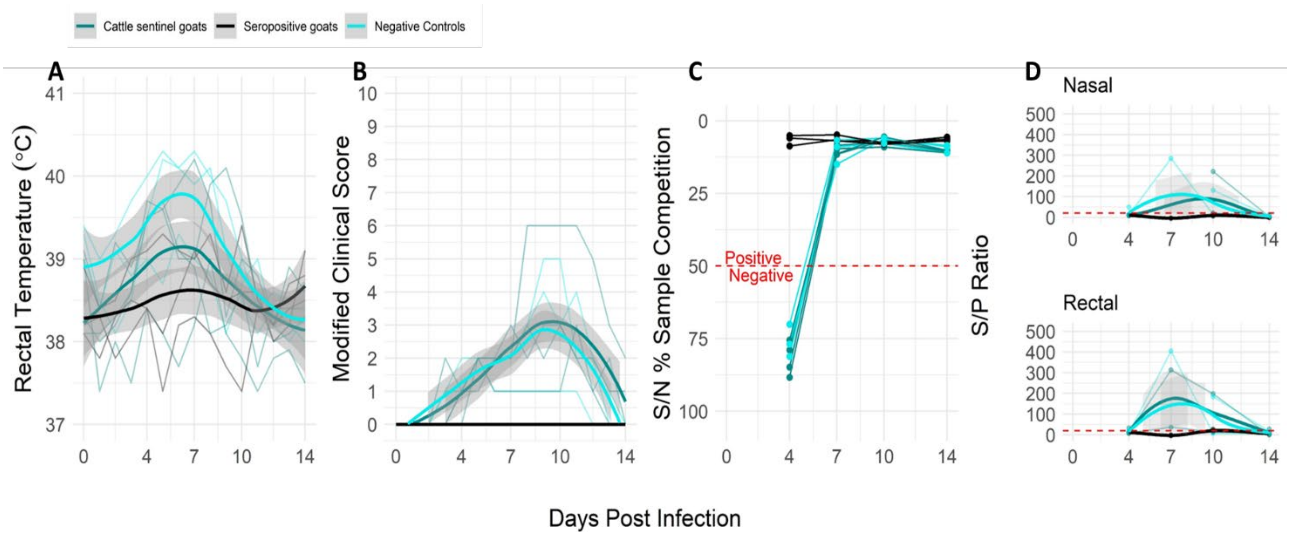

Fig. S9. Clinical, serological, and molecular findings of Trial 3 goats challenged with PPRV. From left to right in each row there is measurements of A. daily rectal temperature, B.daily modified clinical score, C. serology (competitive ELISA), and D. viral RNA from ocular, nasal, and rectal swabs measured on antigen ELISA. No antigen ELISA were run on negative control animals as serology showed they were all negative. Thin lines represent individual animals and bold lines represent smooth local regression (LOESS) curves of all animals in the category inoculated, sentinel). Gray shading indicates 95% confidence bands (t-based approximation). No isolation data was collected during the challenge experiment. No animals died.

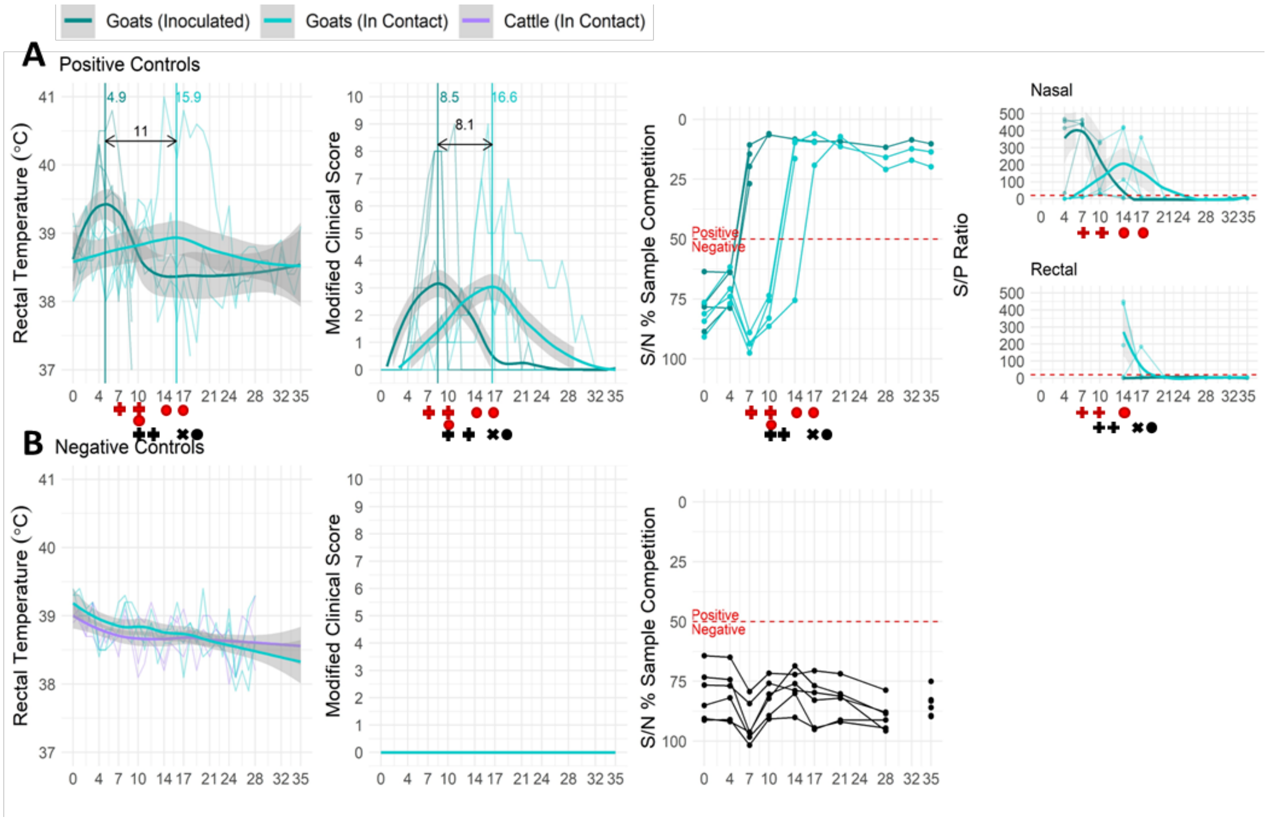

Fig. S10. Trial 3 clinical, serological, and molecular results from positive control goats (A) and clinical and serological data from negative control cattle and goats (B). From left to right in each row there is measurements of daily rectal temperature, daily modified clinical score, serology (competitive ELISA), and viral RNA from ocular, nasal, and rectal swabs measured on antigen ELISA. No antigen ELISA were run on negative control animals as serology showed they were all negative. Thin lines represent individual animals and bold lines represent smooth local regression (LOESS) curves of all animals in the category (inoculated, sentinel). Gray shading indicates 95% confidence bands (t-based approximation). When present, vertical lines indicate day post infection (dpi) of peak value and inter-peak interval (difference in dpi of peak value for each animal group) is indicated in black. Sampling days on which PPRV could be isolated (cross if isolated from inoculated animal, circle for sentinel animal) are indicated with red and dpi with deaths are indicated with black (cross for inoculated animal deaths, circle for sentinel deaths, x for euthanized) along the x-axis. Five positive controls died in total (3 inoculated [2 on dpi 10], 2 sentinel); one of the two sentinels was euthanized (dpi 17).

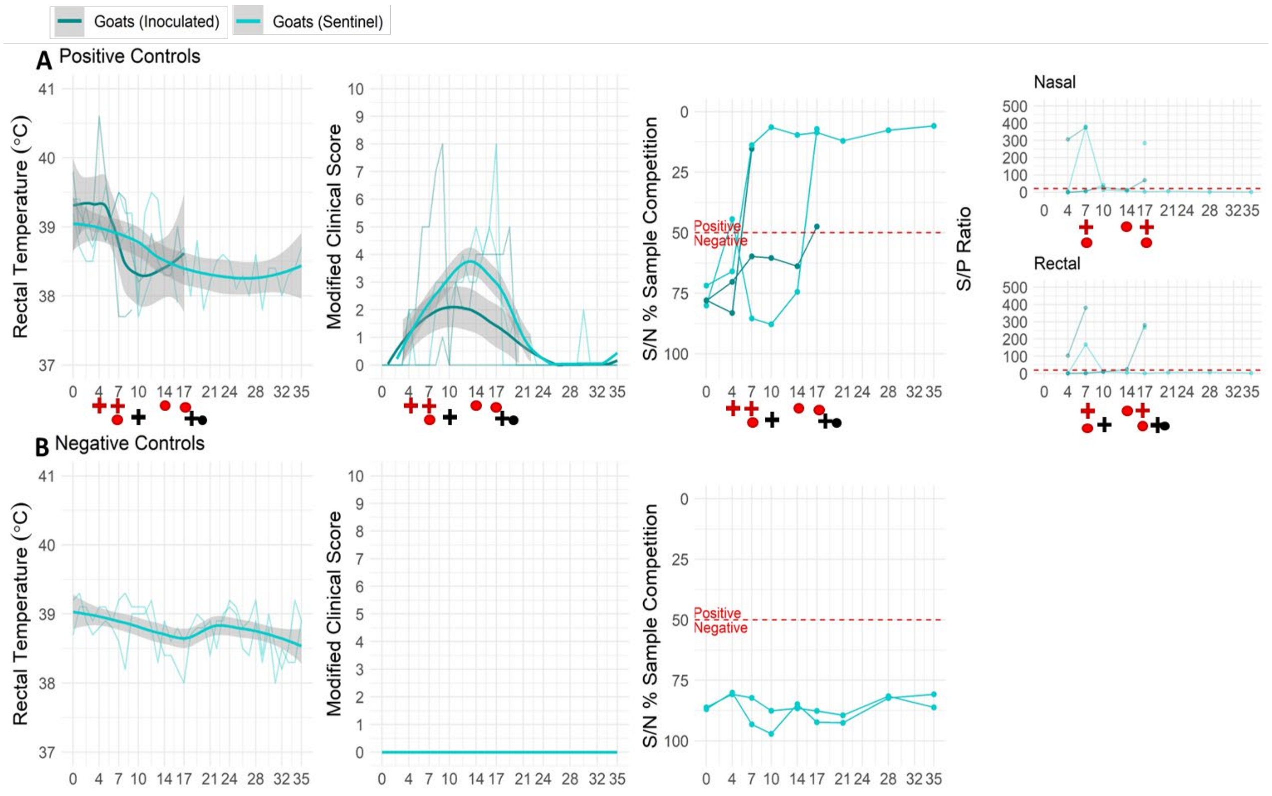

Fig. S11. Trial 4 clinical, serological, and molecular results from positive control goats (A) and clinical and serological data from negative control cattle and goats (B). From left to right in each row there is measurements of daily rectal temperature, daily modified clinical score, serology (competitive ELISA), and viral RNA from ocular, nasal, and rectal swabs measured on antigen ELISA. No antigen ELISA were run on negative control animals as serology showed they were all negative. Thin lines represent individual animals and bold lines represent smooth local regression (LOESS) curves of all animals in the category (inoculated, sentinel). Gray shading indicates 95% confidence bands (t-based approximation). Sampling days on which PPRV could be isolated (cross if isolated from inoculated animal, circle for sentinel animal) are indicated with red and dpi with deaths are indicated with black (cross for inoculated animal deaths, circle for sentinel deaths, x for euthanized) along the x-axis. Four positive controls died in total (2 inoculated, 2 sentinel [dpi18 and dpi 20]).

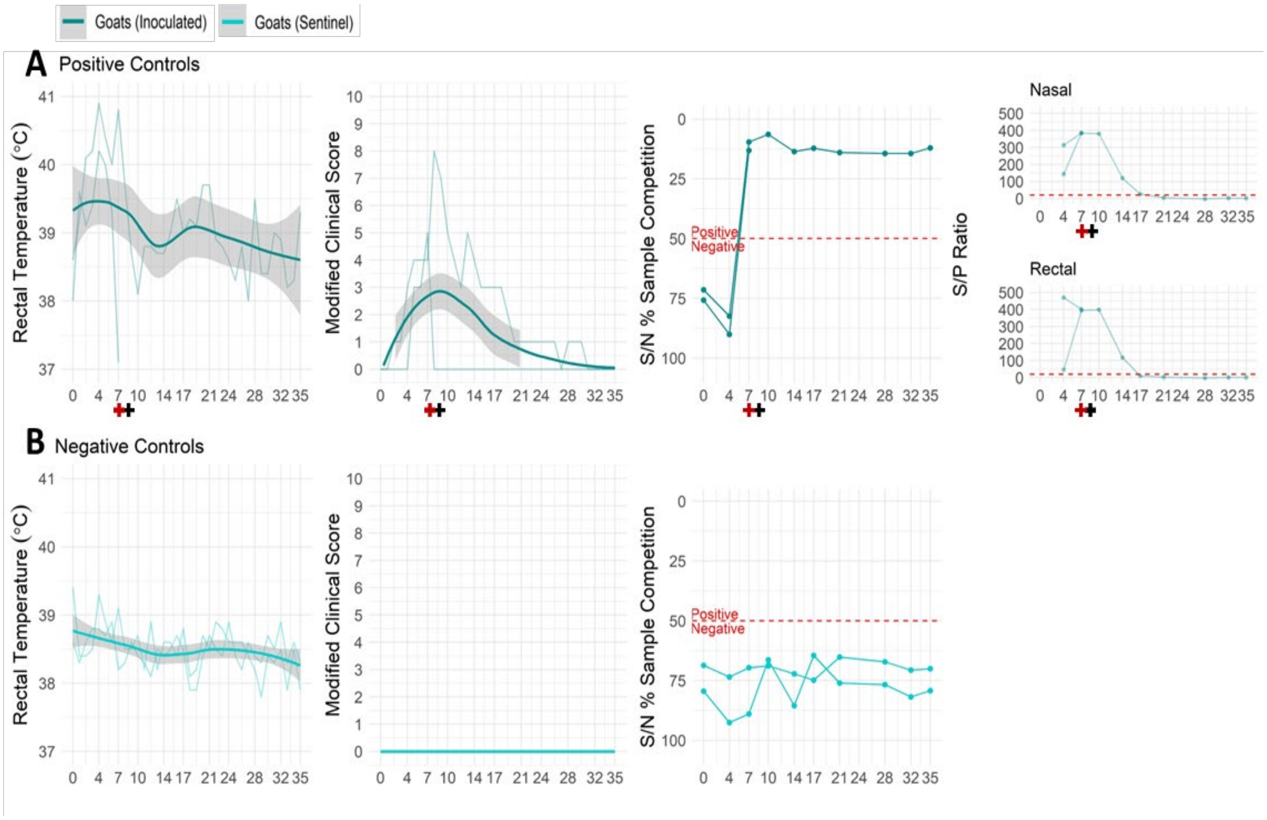

Fig. S12. Trial 5 clinical, serological, and molecular results from positive control goats (A) and clinical and serological data from negative control cattle and goats (B). From left to right in each row there is measurements of daily rectal temperature, daily modified clinical score, serology (competitive ELISA), and viral RNA from ocular, nasal, and rectal swabs measured on antigen ELISA. No antigen ELISA were run on negative control animals as serology showed they were all negative. Thin lines represent individual animals and bold lines represent smooth local regression (LOESS) curves of all animals in the category (inoculated, sentinel). Gray shading indicates 95% confidence bands (t-based approximation). When present, vertical lines indicate day post infection (dpi) of peak value and inter-peak interval (difference in dpi of peak value for each animal group) is indicated in black. Sampling days on which PPRV could be isolated (cross if isolated from inoculated animal, circle for sentinel animal) are indicated with red and dpi with deaths are indicated with black (cross for inoculated animal deaths, circle for sentinel deaths, x for euthanized) along the x-axis. One inoculated positive control died.

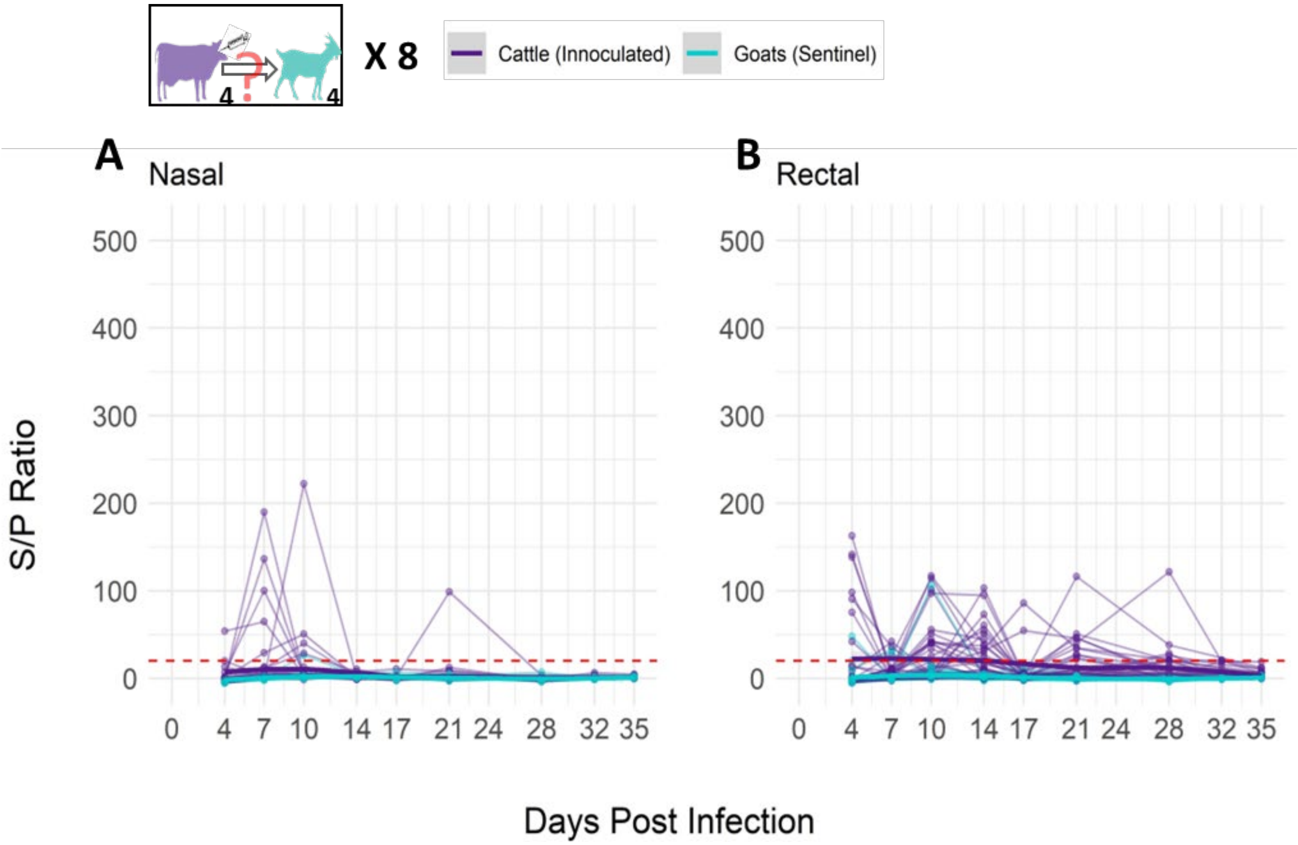

Fig. S13. Trials 3 to 5 antigen ELISA results from nasal (A) and rectal swabs (B) from inoculated cattle and sentinel goats. Thin lines represent individual animals and bold lines represent smooth local regression (LOESS) curves of all animals in the category (inoculated, sentinel). Gray shading indicates 95% confidence bands (t-based approximation).

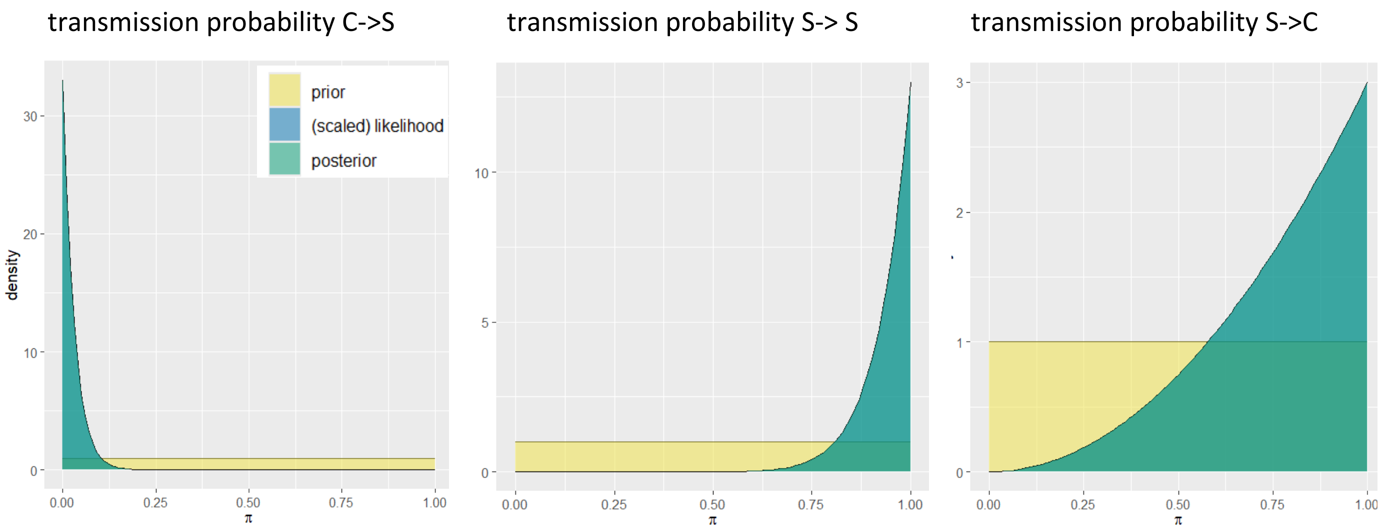

Fig. S14. Posterior distributions of the transmission probabilities observed in the empirical trials, calculated from a beta binomial Bayesian model using bayesrules R package (see Methods, Text S5). Posterior distributions are in green, uniform uninformative priors are in yellow, and the scaled likelihood is in blue (and completely overlapped by the posterior). Cattle (C), small ruminants (S).

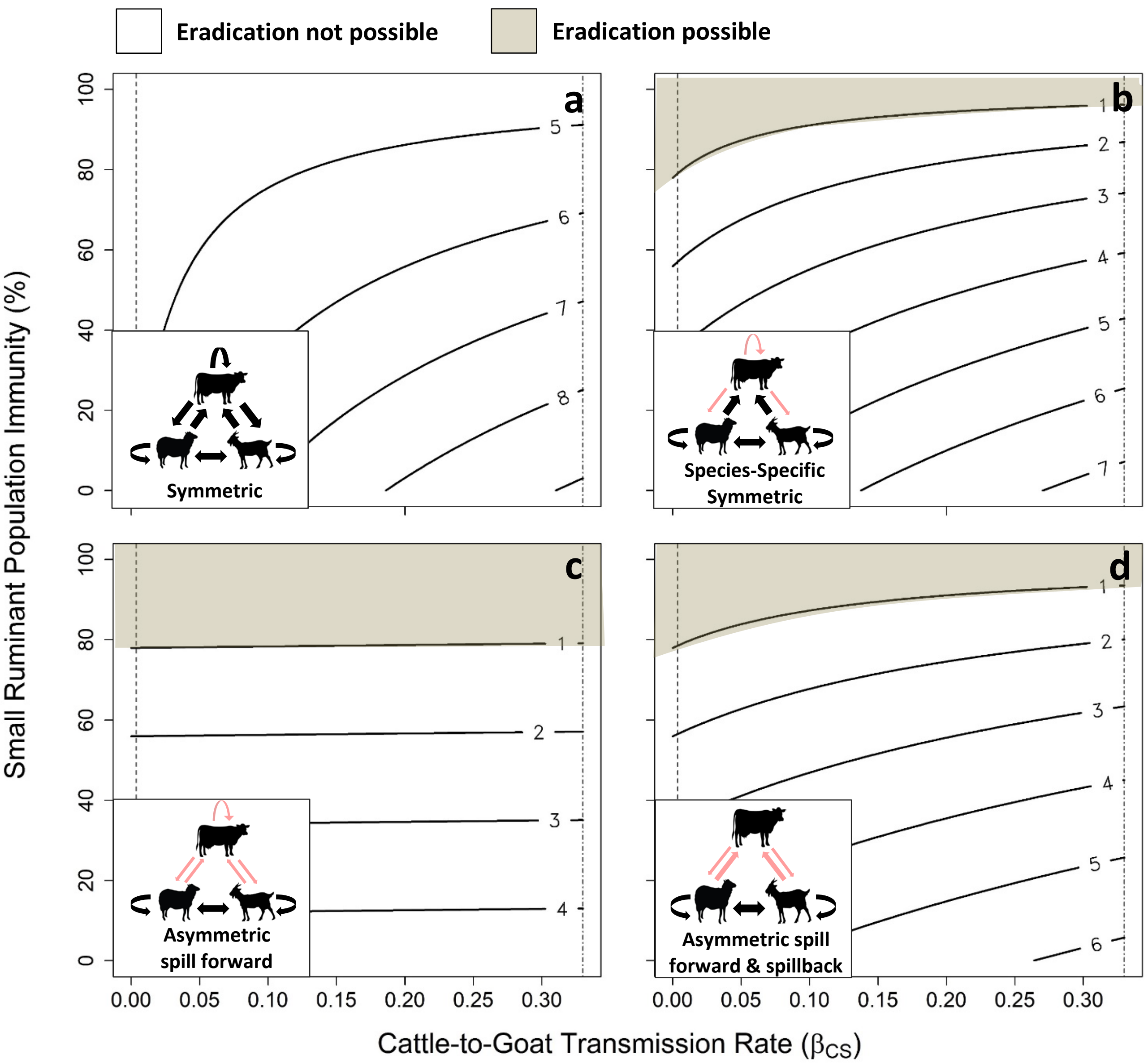

Figure S15. Increasing cattle-to-small ruminant transmission increases the amount of small ruminant vaccination needed in some but not all transmission scenarios. Eradication is possible in scenarios B, C, and D when R_E <1 (i.e. area above the contour labelled "1" in B, C & D and shaded in gray), but is not possible in scenario A. Community R_E values are indicated in the numeric values on labelled contours. For an infectious period of 8 days, the estimated transmission rates for cattle-to-small ruminant and small ruminant-to-small ruminant are shown as dashed (farther left) and dot dashed (farther right) vertical lines, respectively. In scenarios B and D, increasing levels of small ruminant vaccination is indicated, as the rate of cattle-to-small ruminant transmission increases from the observed cattle-to-goat transmission rate to slightly above the goat-to-goat transmission rate observed in the trials. For each transmission scenario, cattle (C) transmission rate to small ruminants (S) varies from 0 to 0.1 (x-axis, gray arrow). In panels B, C, D changes in transmission scenarios relative to panel A are indicated with red arrows or removal of an arrow in the inset figure. Scenario 1 (**A**) explores a symmetric transmission rate for all species ($\beta_{SS} = \beta_{SC} = \beta_{CC} = 3.3 \times 10^{-1}$; β_{CS} varies 0-0.26). Scenario 2 (**B**) explores a symmetric transmission rate within each species ($\beta_{SS} = \beta_{SC} = = 3.3 \times 10^{-1}$; $\beta_{CC} = 3.7 \times 10^{-3}$; β_{CS} varies 0-0.33).). Scenario 3 (**C**) explores asymmetric transmission rate between species

(β_{SS} = 3.3 x 10^{-1}; β_{SC} = β_{CC} =3.7 x 10^{-3}; β_{CS} varies 0-0.33).). Lastly, scenario 4 (**D**) explores asymmetric transmission rate within and between species (β_{SS} = 3.3 x 10^{-1}; β_{SC} = 1.7 x 10^{-1}; β_{CC} = 0; β_{CS} varies 0-0.33).). See Methods, Text S5, Text S6, Table S2 for more information on parameter values selected and model code.

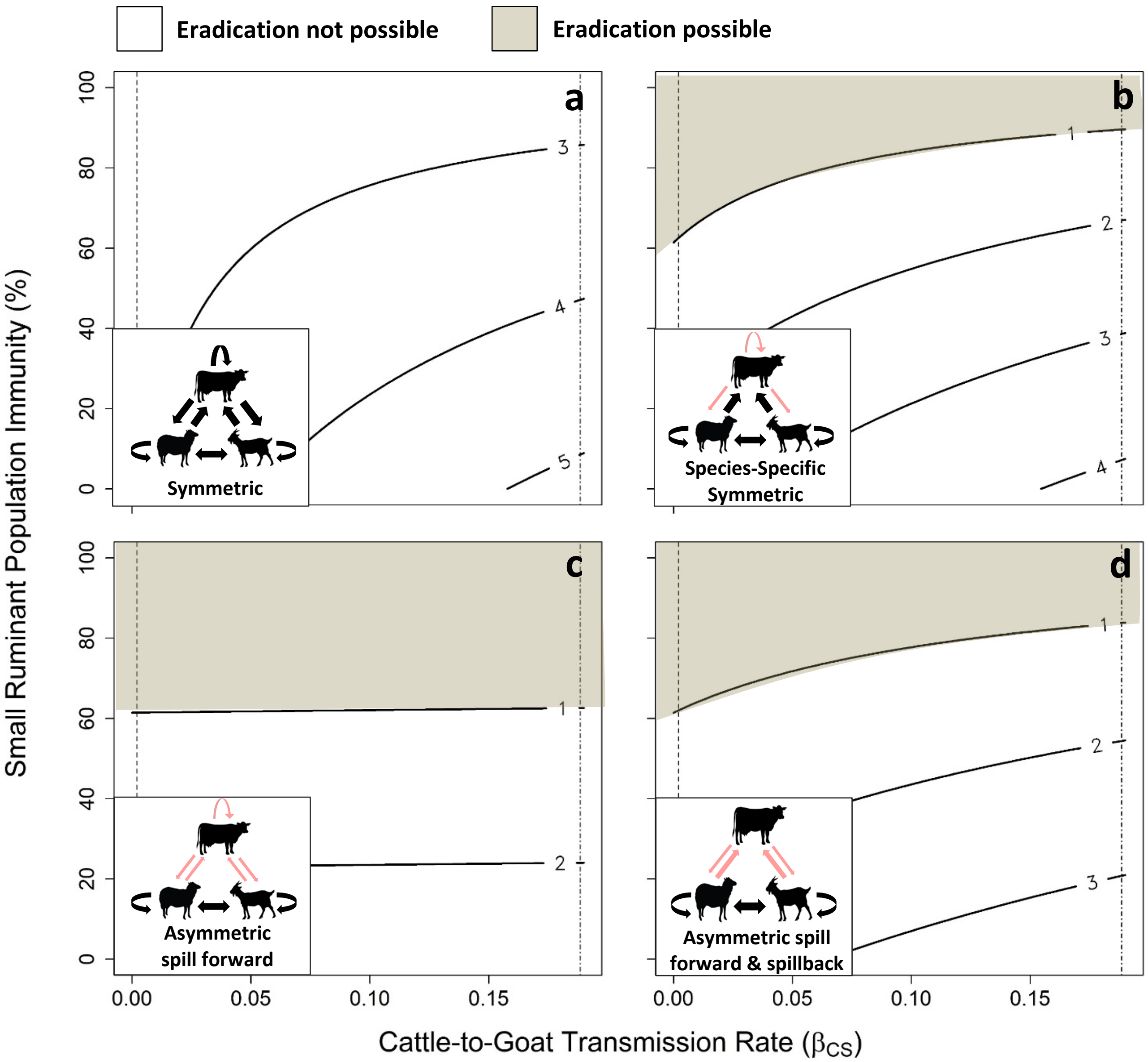

Figure S16. Increasing cattle-to-small ruminant transmission increases the amount of small ruminant vaccination needed in some but not all transmission scenarios. Eradication is possible in scenarios B, C, and D when R_E <1 (i.e. area above the contour labelled "1" in B, C & D and shaded in gray), but is not possible in scenario A. Community R_E values are indicated in the numeric values on labelled contours. For an infectious period of 14 days, the estimated transmission rates for cattle-to-small ruminant and small ruminant-to-small ruminant are shown as dashed (farther left) and dot dashed (farther right) vertical lines, respectively. In scenarios B and D, increasing levels of small ruminant vaccination is indicated, as the rate of cattle-to-small ruminant transmission increases from the observed cattle-to-goat transmission rate to slightly above the goat-to-goat transmission rate observed in the trials. For each transmission scenario, cattle (C) transmission rate to small ruminants (S) varies from 0 to 0.1 (x-axis, gray arrow). In panels B, C, D changes in transmission scenarios relative to panel A are indicated with red arrows or removal of an arrow in the inset figure. Scenario 1 (**A**) explores a symmetric transmission rate for all species ($\beta_{SS} = \beta_{SC} = \beta_{CC} = 1.9 \times 10^{-1}$; β_{CS} varies 0-0.19). Scenario 2 (**B**)

explores a symmetric transmission rate within each species ($\beta_{SS} = \beta_{SC} = 1.9 \times 10^{-1}$; $\beta_{CC} = 2.1 \times 10^{-3}$; β_{CS} varies 0-0.19).). Scenario 3 (**C**) explores asymmetric transmission rate between species ($\beta_{SS} = 1.9 \times 10^{-1}$; $\beta_{SC} = \beta_{CC} = 2.1 \times 10^{-3}$; β_{CS} varies 0-0.19).). Lastly, scenario 4 (**D**) explores asymmetric transmission rate within and between species ($\beta_{SS} = 1.9 \times 10^{-1}$; $\beta_{SC} = 9.9 \times 10^{-2}$; $\beta_{CC} = 0$; β_{CS} varies 0-0.19).). See Methods, Text S5, Text S6, Table S2 for more information on parameter values selected and model code
