# Supplementary material for: Empirical and model-based evidence for a negligible role of cattle in peste des petits ruminants virus transmission and eradication
Source: Commun Biol. 2024 Aug 3;7:937. doi: 10.1038/s42003-024-06619-2 (PMC11297268; doi:10.1038/s42003-024-06619-2)
Supplement: Supplementary file 3 — Reporting Summary [file 42003_2024_6619_MOESM3_ESM.pdf]

nature portfolio

Corresponding author(s): Dr. Vivek Kapur, Dr. Catherine M Herzog

Last updated by author(s): May 18, 2023

Reporting Summary

Nature Portfolio wishes to improve the reproducibility of the work that we publish. This form provides structure for consistency and transparency in reporting. For further information on Nature Portfolio policies, see our Editorial Policies and the Editorial Policy Checklist.

Statistics

For all statistical analyses, confirm that the following items are present in the figure legend, table legend, main text, or Methods section.

n/a	Confirmed	
☐	☒	The exact sample size (*n*) for each experimental group/condition, given as a discrete number and unit of measurement
☐	☒	A statement on whether measurements were taken from distinct samples or whether the same sample was measured repeatedly
☐	☒	The statistical test(s) used AND whether they are one- or two-sided<br>*Only common tests should be described solely by name; describe more complex techniques in the Methods section.*
☒	☐	A description of all covariates tested
☐	☒	A description of any assumptions or corrections, such as tests of normality and adjustment for multiple comparisons
☐	☒	A full description of the statistical parameters including central tendency (e.g. means) or other basic estimates (e.g. regression coefficient) AND variation (e.g. standard deviation) or associated estimates of uncertainty (e.g. confidence intervals)
☒	☐	For null hypothesis testing, the test statistic (e.g. *F*, *t*, *r*) with confidence intervals, effect sizes, degrees of freedom and *P* value noted<br>*Give P values as exact values whenever suitable.*
☒	☐	For Bayesian analysis, information on the choice of priors and Markov chain Monte Carlo settings
☒	☐	For hierarchical and complex designs, identification of the appropriate level for tests and full reporting of outcomes
☒	☐	Estimates of effect sizes (e.g. Cohen's *d*, Pearson's *r*), indicating how they were calculated

Our web collection on statistics for biologists contains articles on many of the points above.

Software and code

Policy information about availability of computer code

Data collection	Paper, MS Excel
Data analysis	R statistical software 4.1.3

For manuscripts utilizing custom algorithms or software that are central to the research but not yet described in published literature, software must be made available to editors and reviewers. We strongly encourage code deposition in a community repository (e.g. GitHub). See the Nature Portfolio guidelines for submitting code & software for further information.

Data

Policy information about availability of data

All manuscripts must include a data availability statement. This statement should provide the following information, where applicable:

- Accession codes, unique identifiers, or web links for publicly available datasets
- A description of any restrictions on data availability
- For clinical datasets or third party data, please ensure that the statement adheres to our policy

The dataset used in this study will be made available upon request to the corresponding author. Whole genome sequence information for PPRV/Ethiopia/Habru/2014 passage 4 can be found at Genbank accession number: ON110960

Research involving human participants, their data, or biological material

Policy information about studies with human participants or human data. See also policy information about sex, gender (identity/presentation), and sexual orientation and race, ethnicity and racism.

Reporting on sex and gender	NA
Reporting on race, ethnicity, or other socially relevant groupings	NA
Population characteristics	NA
Recruitment	NA
Ethics oversight	NA

Note that full information on the approval of the study protocol must also be provided in the manuscript.

Field-specific reporting

Please select the one below that is the best fit for your research. If you are not sure, read the appropriate sections before making your selection.

☒ Life sciences ☐ Behavioural & social sciences ☐ Ecological, evolutionary & environmental sciences

For a reference copy of the document with all sections, see nature.com/documents/nr-reporting-summary-flat.pdf

Life sciences study design

All studies must disclose on these points even when the disclosure is negative.

Sample size	Description and code given in Supplemental Text S4.
Data exclusions	No data were excluded from analyses
Replication	All blood and swab samples were tested in duplicate (for serology, AgELISA RT-qPCR).
Randomization	Purchased animals were randomly selected to be in the same barn, choice of barn was randomly selected, and which barns were experimental and control barns were randomly selected.
Blinding	Investigators were not blinded.

Reporting for specific materials, systems and methods

We require information from authors about some types of materials, experimental systems and methods used in many studies. Here, indicate whether each material, system or method listed is relevant to your study. If you are not sure if a list item applies to your research, read the appropriate section before selecting a response.

Materials & experimental systems

n/a	Involved in the study	
☒	☐	Antibodies
☐	☒	Eukaryotic cell lines
☒	☐	Palaeontology and archaeology
☐	☒	Animals and other organisms
☒	☐	Clinical data
☒	☐	Dual use research of concern
☒	☐	Plants

Methods

n/a	Involved in the study	
☒	☐	ChIP-seq
☒	☐	Flow cytometry
☒	☐	MRI-based neuroimaging

Eukaryotic cell lines

Policy information about cell lines and Sex and Gender in Research

Cell line source(s)	The Pirbright Institute, United Kingdom

Authentication	Expression of SLAM was functionally confirmed, no genetic characterization completed, cells did not produce type I interferon
Mycoplasma contamination	Confirmed negative by Pirbright cell culture service and individual lab via immunofluorescence staining.
Commonly misidentified lines (See ICLAC register)	*Name any commonly misidentified cell lines used in the study and provide a rationale for their use.*

Animals and other research organisms

Policy information about studies involving animals; ARRIVE guidelines recommended for reporting animal research, and Sex and Gender in Research

Laboratory animals	Did not involve laboratory animals.
Wild animals	Local breeds of sheep (Ovis aries), goats (Capra aegagrus hircus), and Zebu calves (Bos taurus indicus) were purchased from markets within a 200 km radius of AHI (Sebeta, Ethiopia). There was no reported history of PPRV vaccination in any animal purchased. Animals were of both sexes and ranged in age from 6 months to 1.5 years. Once seronegative status was confirmed, animals were moved into the six-barn experimental facility for acclimatization. Water and hay were provided ad libitum and refreshed daily. On each day, animals were fed, the barn cleaned of old feed and waste, the rectal temperatures of all animals was determined, and animals were monitored for clinical signs.
Reporting on sex	Sex was recorded for each animal but was not a variable for stratification or comparison in study design.
Field-collected samples	Nasal, ocular, and rectal swabs were collected in duplicate from the animal testing facility and sent directly to the molecular and cell culture laboratories for processing. Both locations were in the same campus.
Ethics oversight	The study protocol ARSERC/EC/001/17/04/2019 was reviewed and approved on May 3, 2019 by the Animal Health Institute's (AHI) Animal Research Scientific Ethics Review Committee (ARSERC)

Note that full information on the approval of the study protocol must also be provided in the manuscript.
